# Prevalence of dental anomalies in Indian population

**DOI:** 10.4317/jced.51119

**Published:** 2013-10-01

**Authors:** Santosh Patil, Bharati Doni, Sumita Kaswan, Farzan Rahman

**Affiliations:** 1 Dept of Oral medicine and radiology, Jodhpur Dental College, Jodhpur National University, Jodhpur (Raj), India; 2 Dept of Oral medicine and radiology, NIMS, Jaipur (Raj), India; 3 Dept of Conservative Dentistry and Endodontics, Jodhpur Dental College, Jodhpur National University, Jodhpur (Raj), India; 4 Dept of Oral Pathology and Microbiology, Jaipur Dental College and Hospital, Jaipur (Raj), India

## Abstract

Objectives: Developmental anomalies of the dentition are not infrequently observed by the dental practitioner. The aim of the present study was to determine the prevalence of dental anomalies in the Indian population. Study Design: A retrospective study of 4133 panoramic radiographs of patients, who attended the Department of Oral Medicine and Radiology, Jodhpur Dental College General Hospital between September 2008 to December 2012 was done. The ages of the patients ranged from 13 to 38 years with a mean age of 21.8 years. The orthopantomographs (OPGs) and dental records were examined for any unusual finding such as congenitally missing teeth, impactions, ectopic eruption, supernumerary teeth, odontoma, dilacerations, taurodontism, dens in dente, germination and fusion, among others. Results: 1519 (36.7%) patients had at least one dental anomaly. The congenitally missing teeth 673 (16.3%) had the highest prevalence, followed by impacted teeth 641 (15.5%), supernumerary teeth 51 (1.2%) and microdontia 41 (1.0%). Other anomalies were found at lower prevalence ranging from transposition 7 (0.1%) to ectopic eruption 30 (0.7%). Conclusion: The most prevalent anomaly in the Indian population was congenitally missing teeth (16.3%), and the second frequent anomaly was impacted teeth (15.5%), whereas, macrodontia, odontoma and transposition were the least frequent anomalies, with a prevalence of 0.2%, 0.2% and 0.1% respectively. While the overall prevalence of these anomalies may be low, the early diagnosis is imperative for the patient management and treatment planning.

** Key words:**Dental anomaly, prevalence, panoramic radiography.

## Introduction

Developmental dental anomalies are an important category of dental symptomatology. Their incidence and degree of expression in different population groups can provide important information for phylogenic and genetic studies and help the understanding of variations within and between the different populations. The various dental anomalies of the dentition are frequently observed in the dental clinic. However, compared to the more common oral diseases such as dental caries and periodontal diseases, these anomalies account for a relatively low number, but can pose a problem during treatment planning. They present with malocclusion, esthetic and functional problem, and possible disposition to other oral diseases. Hence, their clinical management is usually complicated (1).

Dental anomalies in tooth number, shape, structure, and position may result in problems in arch length and occlusion, which may greatly influence treatment planning for the orthodontists. The etiology of these conditions is usually suggested to be either due to genetic factors in addition to some etiological events during the prenatal and postnatal development periods and environmental and pathological factors (2-4). According to Sarnat and Schour (5), the growing tooth acts as a biological recorder which provides a precise and permanent record of variations and fluctuations in the tooth matrix and its mineralization. These anomalies may be involving one tooth or generalized to involve all the teeth or they may be present as a part of any systemic disorders or syndromes (1). One or more dental anomalies can often be observed in the same patient. Studies on the patterns of association among seven types of dental anomalies in an untreated orthodontic population aged 7 to 14 years found a significant reciprocal association among 5 of the anomalies that suggests a common genetic origin. It was found that 34% of the patients with conical-shaped upper lateral incisors had palatally displaced canine (6).

Radiographic and clinical examination may reveal these dental anomalies. The aim of the present study was to determine the presence of various developmental anomalies through examination of panoramic radiographs in the Indian population.

## Material and Methods

The panoramic radiographs of 4133 patients attending the Department of Oral Medicine and Radiology, Jodhpur Dental College General Hospital between September 2008 to December 2012 were examined for the presence of various dental anomalies. Ethical committee clearance was obtained from the concerned authority. The ages of the patients ranged from 13 to 38 years with a mean of 21.8 years. All panoramic radiographs were taken with the Dentsply Gendex Orthoralix 9200 (Dentsply Asia, Milford, US), and the magnification factor was 1.23. All reported measurements were adjusted according to this factor. The panoramic radiographs were examined on standard light boxes, under good lighting conditions, standardized screen brightness and resolution to determine the dental anomalies. Patients’ dental records and radiographs were examined in order to detect the following dental anomalies: congenitally missing teeth, impactions, ectopic eruption, supernumerary teeth, germination, fusion, dilacerations, taurodontism, dens in dente, microdontia, macrodontia, and any other unusual finding that can be assessed with OPG. After the examination of the patient records, patients who exhibited any pathological conditions, trauma or fracture of the jaw that might have affected the normal growth of permanent dentition or any hereditary diseases or syndromes were excluded from the study.

## Results

The study comprised of 2145 males (51.9%) and 1988 females (48.1%) with an age range of 13 to 38 years with a mean age of 21.8 years. 1519 patients with a prevalence of 36.7% had at least one dental anomaly.  Table 1 shows the distribution of patients according to gender and the prevalence of the dental anomalies present. The congenitally missing teeth 673 (16.3%) had the highest prevalence. 513 (12.4%) of patients had missing third molar, 63 (1.6%) had missing premolar and 18 (0.4%) had a canine that was missing. A total of 641 impacted teeth were found with a prevalence of 15.5% and third molars (9.7%) were the most commonly impacted tooth. The supernumerary teeth were found in 51 patients with a prevalence of 1.2% and most common supernumerary tooth was mesiodens. The prevalence of microdontia was 1.0%. The prevalence of other anomalies was as follows: ectopic eruption 0.7%, dilacerations 0.5%, dens in dente 0.4%, taurodontism 0.4%, odontoma 0.2%, macrodontia 0.2% and transposition 0.1%. None of the patients showed fusion and germination.

**Table 1:**
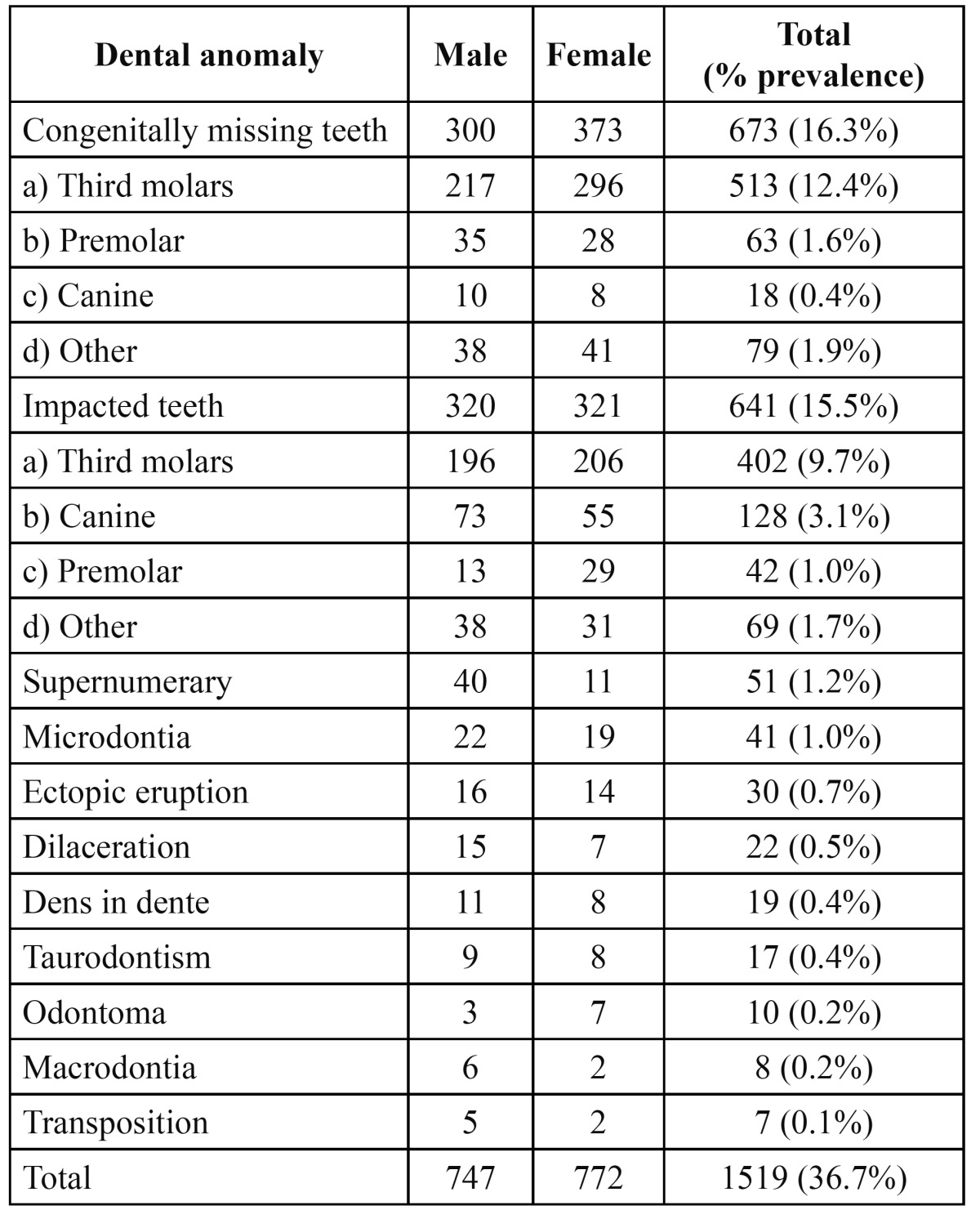
Distribution of patients with different dental anomalies according to gender.

## Discussion

Dental anomalies may be expressed with mild developmental delay to the most severe tooth agenesis; dental anomalies may be expressed as microdontia, changes in dental shape, structure and ectopias (7). The etiology of dental anomalies of number, size, position, as well as timing of development, have been suggested to be genetic and hereditary, as derived from studies in families, monozygotic twins, and from the frequent observation of associations of certain dental anomalies (6,8). These dental anomalies can complicate orthodontic treatment if not considered; therefore, their presence should be thoroughly investigated during diagnosis and carefully considered during treatment planning. Many epidemiological surveys have been done in the recent past in different parts of the world to determine the prevalence of dental anomalies (1,9,10). The results of these studies have shown that variations in the prevalence of dental anomalies could be due to regional and racial differences. The prevalence of some abnormalities such as hypodontia and supernumeraries may have been underestimated in few studies as these studies were conducted without any radiographic assessment. This study was done to detect the prevalence of dental anomalies in the Indian population using the panoramic radiographs of the patients.

The prevalence of dental anomalies reported in this study was quite high due to a large number of the anomalies of the wisdom teeth which had a prevalence of 36.7%. A high prevalence of congenitally missing and impacted third molar teeth is reported in this study similar to the results reported in earlier studies. The present findings showed that the incidence of impaction was found to be 15.5%, which is higher than the findings of the previous studies (11-13). Afify and Zawawi (1) reported a higher prevalence of impacted teeth (21.2%). In this study, canines were the most commonly impacted teeth, excluding third molars with the prevalence of 3.1%, which is much lower than the studies of Fardi et al. (14) who reported a prevalence of 8.8% in the Greek population. In a similar study, 4898 Saudi patients aged 13 years and older were examined, who showed a prevalence of 3.6% with at least one impacted cuspid (15). Another study that analyzed 1858 patients of the 11-18 year age group presented for orthodontic treatment, revealed 101 cases of impacted canines with a prevalence of 5.43% (16). Aydin et al. (17) and Afify and Zawawi (1) reported an incidence of 3.58% and 3.3% which was in line with the findings of the present study. One limitation to the findings of the present study is that the impactions were not classified as partial or fully impacted and the angulation of impaction was also not taken into consideration. The Japanese have shown to have the lowest frequency as reported in the literature, where the anomaly occurred in only 0.27% of the study population (18). Similar to these findings, study of a large series of full mouth dental radiographs in the USA revealed a figure of 0.92% (19). While Brin et al. (20) in their study of an Israeli population, found a level of 1.5%.

Mandibular canine impaction is rarely seen and there are limited studies revealing its frequency of occurrence. Impacted canines of the mandible are very rare in occurrence. In the study by Shah et al. (11), 8 unerupted mandibular canines were found in 7886 individuals, and in another study only 11 impacted mandibular canines were found in 5000 individuals, resulting in an incidence of 0.10% (12). Very few studies have been done regarding impacted premolars. It has been concluded from these studies that premolar impaction is rare, with the prevalence ranging from 2.1-2.7% (13,19,21). The results of the present study are however lower, with a prevalence of 1.0%. To determine the actual prevalence of tooth impaction, a representative and randomized sample of the general population is required. Here, radiographic examination from specific populations seems to be the most common practical approach, which will inevitably involve the risk of bias in the data analysis.

Supernumerary teeth are a frequent finding in dental practice. Supernumerary teeth or hyperdontia describes an excess in tooth number. The prevalence of hyperdontia is reported to lie between 1-3% in permanent dentition and is rarely seen in the primary dentition. The aetiology is unknown, and several theories have been suggested. The prevalence of 1.2% of supernumerary impacted teeth as reported in the present study falls within the range of 0.1-3.8% as reported earlier (14,22). Bäckman and Wahlin (22) found 14 cases with one supernumerary tooth in a study in the Caucasian population. They also noted that the majority of the supernumerary teeth were mesiodens, similar to the present study. Another study of 2,393 Saudi Arabian children found the prevalence of supernumerary tooth to be 0.5% (9). The prevalence of supernumerary teeth in the western region of Saudi Arabia was reported to be 0.3% (1). Most supernumerary teeth are impacted and asymptomatic and diagnosed incidentally during radiographic examinations. Panoramic radiograph is thus essential for the early detection of supernumerary teeth. However, clinical complications are not uncommon in patients with supernumerary teeth. Tooth displacement and failure of eruption are the most frequently seen complications (23).

Dental morphology is only one of several factors that may be involved in the etiology of crowding or spacing in the dental arch. Macrodontia is very much less common than microdontia (24). The prevalence of macrodontia and microdontia in the current study was in similar when compared with other studies (0.2% and 1.0%, respectively) (24,25). The most common microdontia was of the maxillary lateral incisor (24,26). Crown dilaceration of a permanent tooth constitutes 3% of traumatic injuries to developing tooth (27) and usually involves the maxillary incisors (28). There were 22 dilaceration cases in this study and history of trauma was obtained in all cases. The prevalence of dilacerations was reported to be 1.1% by Afify and Zawawi (1), which is in line with the present study. Odontoma was noted in 0.2% of the subjects, similar to the findings of Afify and Zawawi (0.1%) (1), and was seen mostly in maxillary anterior and the 3rd molar regions. They prevent the eruption of a tooth and have to be surgically removed. Teeth transposition is a rare eruption anomaly that involves the permanent dentition (incidence 0.3-0.4%) and are more frequently seen in the maxilla (29). Transposition may occur with other anomalies, such as aplasia, peg-shaped lateral incisor and deciduous teeth retention (30).

The present study showed that the prevalence of various dental anomalies shows variations from other similar studies. The dissimilarities may be attributed to the sample selection, method of the study and area of patient selection, which suggest racial and genetic differences. Early detection of dental development anomalies is very important, as they may lead to many unseen complications. Whether the prevalence of dental anomaly leads to any orthodontic problem has however, not been fully understood. Diagnosis could be made at the radiological level; the earlier the diagnosis, the less risks related to treatment.

While the overall prevalence of each of these anomalies in the dental clinic or population group may be low, their presence may, in some cases create a management problem or complicate treatment options for patients. Careful diagnosis simplifies the treatment plan and reduces complications.
